# Diffuse Alveolar Hemorrhage as a First Presentation for Acute Mitral Regurgitation: Case Report

**DOI:** 10.7759/cureus.109690

**Published:** 2026-05-26

**Authors:** Kawthar A Alwadi, Husain A Habib, Lujayn F Juma, Jaafar Alsadeq M Laith, Eman M AlArab

**Affiliations:** 1 Department of Internal Medicine/Respiratory Unit, Salmaniya Medical Complex, Manama, BHR; 2 Faculty of Medicine, Mansoura University, Mansoura, EGY

**Keywords:** acute respiratory distress syndrome, case report, community-acquired pneumonia, diffuse alveolar hemorrhage, mitral regurgitation

## Abstract

Diffuse alveolar hemorrhage (DAH) is a medical emergency that is known to be caused by systemic vasculitis, autoimmune conditions, coagulation disorders, and toxins. While it can be caused by chronic mitral regurgitation (MR), it has a rare association with acute MR. We report the case of a patient presenting with DAH as the first sign of acute MR. A 58-year-old man with type 2 diabetes mellitus and no cardiac history except for hypertension presented with dyspnea and cough with pink-stained white sputum for two days. On examination, bilateral crepitations and a pansystolic murmur were noticed. N-terminal pro-B-type natriuretic peptide (NT-proBNP) was 165 ng/L, while high-resolution computed tomography (HRCT) showed diffuse bilateral ground-glass opacities with a crazy paving pattern. Transesophageal echocardiography (TEE) showed preserved left ventricular ejection fraction (>60%) and confirmed moderate-to-severe eccentric acute MR due to posterior leaflet prolapse with rupture of marginal chordae tendineae. Bronchoalveolar lavage (BAL) confirmed alveolar hemorrhage and established the diagnosis of DAH, which initially mimicked severe respiratory infection due to the presence of pink-stained sputum and bilateral infiltrates. Following stabilization, the patient was scheduled for mitral valve replacement. This case illustrates the importance of considering cardiac etiologies and performing early echocardiography in patients presenting with unexplained DAH, as these etiologies can mimic common causes of DAH.

## Introduction

Diffuse alveolar hemorrhage (DAH) is a severe condition characterized by bleeding into the alveolar space, resulting in a defect in gas exchange and hypoxemia, which can potentially progress to acute respiratory distress syndrome (ARDS) [1]. Differential diagnosis is broad and includes common causes such as systemic vasculitis, autoimmune conditions, coagulopathies, bone marrow and lung transplantations, and toxins and drugs. While it can be caused by chronic mitral regurgitation (MR), it has a rare association with acute MR. DAH is clinically suspected in patients presenting with acute-onset hemoptysis, fever, respiratory failure, and new-onset radiographic infiltrates, often accompanied by a precipitous drop in hemoglobin; however, it is definitively established by the increase in blood return in bronchoalveolar lavage (BAL) during bronchoscopy [2].

In acute MR, sudden regurgitation of left ventricular blood into the left atrium rapidly increases the left atrial pressure, resulting in an abrupt increase in pulmonary venous and capillary pressures. Because the left atrium has limited time to adapt to the sudden pressure overload, the pulmonary hydrostatic pressure will be markedly elevated. The resulting increase in capillary pressure will eventually cause the disruption of the alveolar capillary-membrane, allowing leakage of blood, plasma, and protein into the alveolar spaces, ultimately leading to pulmonary edema, impaired gas exchange, hypoxemia, and hemoptysis [3].

We report a case of a patient who presented with DAH as the initial presentation of acute MR. This case highlights and emphasizes the importance of an early echocardiogram to identify or rule out cardiac etiologies in the management of patients with unexplained DAH.

## Case presentation

A 58-year-old man with a history of type 2 diabetes and hypertension was referred to the emergency department in Salmaniya Medical Complex, complaining of progressive dyspnea for two days associated with a cough with pink-stained white sputum and fever. The patient denied recent sick contacts, chest pain, syncope, or decreased exercise tolerance. A comprehensive review of systems was otherwise unremarkable.

On physical examination, the patient was conscious, awake, and oriented; however, his clinical condition was critical, as evidenced by severe respiratory distress requiring a non-rebreather mask (NRBM) at 15 L/min. He complained of orthopnea, indicating significant cardiorespiratory compromise. Auscultation revealed bilateral crepitations, suggestive of pulmonary edema, along with a pansystolic murmur at the mitral area radiating to the axilla, raising concern for acute mitral valve pathology contributing to his deterioration. Despite the absence of lower limb edema, his oxygen saturation remained dangerously low at 74%, even with high-flow oxygen support, highlighting severe hypoxemia and imminent risk of respiratory failure. Vital signs showed borderline hemodynamic stability (BP 121/63 mmHg, pulse 101 BPM), but the persistent hypoxia and respiratory findings indicate a potentially life-threatening condition requiring urgent intervention.

At presentation, a complete blood count showed leukocytosis with neutrophilia of about 13.91 x 10⁹/L and 12 x 10⁹/L, respectively, hemoglobin of 13.7 g/dL, and platelets of 346 x10⁹/L. Moreover, a notable elevation in serum creatinine (145.29 µmol/L) and urea (9.1 mmol/L) has been observed. Other essential laboratory tests were within a reference range. Inflammatory markers revealed that erythrocyte sedimentation rate (ESR) was 36 mm/hour, C-reactive protein (CRP) was 190 mg/L, and D-dimer was 3.26 mg/L (Table 1). Cardiac enzymes demonstrated an increase in creatine kinase (431 U/L) and urgent N-terminal pro-B-type natriuretic peptide (NT-proBNP) (165 ng/L), while a normal level of troponin-I was 0.01 ng/ml. Arterial blood gases (ABG) were obtained while the patient was on NRBM, and the results were pH 7.275, pCO2: 29.5 mmHg, pO2: 54 mmHg, sO2: 87%, HCO3: 13.5 mEq/L (Table 2).

**Table 1:** Initial laboratory results.

Test	Result	Reference range
White blood cells count	13.91 x 10⁹/L	3.6-9.6 x 10⁹/L
Neutrophils absolute count	12 x 10⁹/L	2.3-8.1 x 10⁹/L
Hemoglobin	13.7 g/dL	12-14.5 g/dL
Platelets	346 x 10⁹/L	150-400 x 10⁹/L
Serum creatinine	145.29 µmol/L	55-96 µmol/L
Urea	9.1 mmol/L	3.2-8.2 mmol/L
Erythrocyte sedimentation rate (ESR)	36 mm/hour	≤20 mm/hour
C-reactive protein (CRP)	190 mg/L	0-3 mg/L
D-dimer	3.26 mg/L	0.09-0.33 mg/L
Creatine kinase	431 U/L	46-171 U/L
N-terminal pro-B-type natriuretic peptide (NT-proBNP)	165 ng/L	≤125 ng/L
Troponin-I	0.01 ng/ml	<1.5 ng/ml

**Table 2:** Results of the arterial blood gases (ABG) on a non-rebreather mask.

Test	Result	Reference range
pH	7.275	7.35-7.45
PaCO2	29.5 mmHg	35-45 mmHg
PaO2	54 mmHg	80-100 mmHg
SaO2	87%	94-100 %
HCO3	13.5 mEq/L	22-26 mEq/L

Electrocardiogram was normal with no ischemic changes. Transthoracic echocardiogram (TTE) showed suspicion of acute MR. So, TEE was scheduled for the patient to confirm the diagnosis.

According to the previously mentioned results, the patient had leukocytosis with prominent neutrophils. Elevated serum creatinine and slightly elevated urea levels indicated a risk of acute kidney injury. Moreover, high inflammatory markers with a slight rise in cardiac enzymes. An acute non-compensated respiratory acidosis was detected in ABG, with suspension of MR in TTE. All these abnormalities indicate a critical and life-threatening situation.

As a radiological assessment, the chest X-ray showed bilateral patchy ground-glass opacities that are asymmetrically more in the left side, with preserved costophrenic angles, and absence of pulmonary cephalization and Kerley B lines, raising suspicion for DAH (Figure 1).

**Figure 1:**
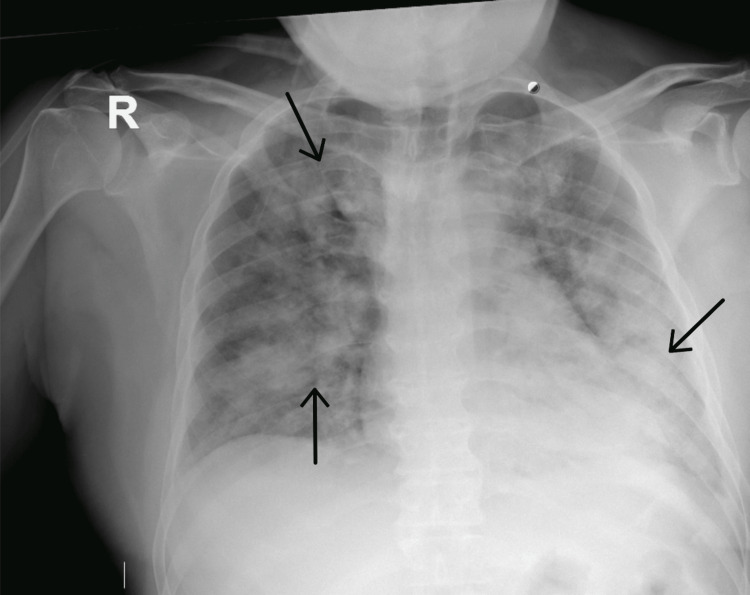
The chest X-ray showed bilateral opacity with a patchy, ill-defined pattern.

The patient was given both glyceryl trinitrate and furosemide as needed for stabilization of acute MR. Due to suspected community-acquired pneumonia (CAP), a five-day course of azithromycin, meropenem, and linezolid was initiated (Table 3).

**Table 3:** Medication sheet.

Drug’s name	Dose	Route	Frequency	Indications
Glyceryl trinitrate	0.5 mg	Sublingual	As needed, with chest pain	Stabilization for acute mitral regurgitation
Furosemide 20 mg/2ml	600-800 mg	IV injection	As needed	
Azithromycin	500 mg	Oral	Once daily	Antibiotics for the suspicion of community-acquired pneumonia
Meropenem	1000 mg	IV injection	Every 12 hours	
Linezolid 600 mg/300 ml	600 mg	IV infusion	Every 12 hours	

He was admitted to the intensive care unit, where he became hypotensive, so glyceryl trinitrate was discontinued, and dopamine was initiated for inotropic support. Additionally, due to respiratory distress, he was intubated and placed on Assist-Control/Volume Control (AC/VC) mechanical ventilation.

Further lab investigations showed a fall in hemoglobin level (9.4 g/dL), a severe impairment in the estimated glomerular filtration rate (eGFR) (26 mL/min/1.73 m²), and an elevation of both serum creatinine and urea levels (228.34 µmol/L, 16.8 mmol/L, respectively), that normalized within five days.

Meanwhile, the autoimmune and vasculitis panel, including anti-double-stranded DNA (anti-dsDNA), antinuclear antibody (ANA), anti-glomerular basement membrane (anti-GBM) antibody, antineutrophil cytoplasmic antibody (ANCA), C3 and C4 complement levels, and rheumatoid factor (RF), was normal. Tests for influenza A and B viruses, human parainfluenza virus, severe acute respiratory syndrome coronavirus 2 (SARS-CoV-2), human adenovirus, respiratory syncytial virus, and *Chlamydophila pneumoniae* were negative. For microbiology, peripheral blood, endotracheal secretions, and urine midstream samples were sterile, with few WBC detected in endotracheal secretions.

The chest high-resolution computed tomography (HRCT) showed diffused bilateral asymmetric ground-glass alveolar opacities in both lungs, with a crazy paving pattern, and the left side predominant. Also, there was bilateral mild-to-moderate pleural effusion (Figure 2).

**Figure 2:**
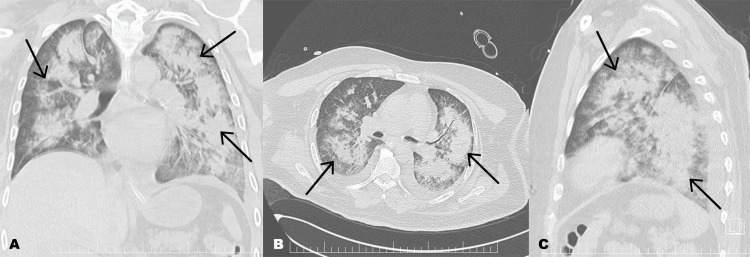
Chest high-resolution computed tomography suggested diffuse alveolar hemorrhage. (A) Coronal view; (B) Axial view-middle lobe; (C) Sagittal view.

TEE confirmed the presence of eccentric moderate-to-severe MR (Figure 3). It demonstrated ruptured primary marginal chordae tendineae at the site of P2 and P3, resulting in a posterior leaflet prolapse. The left atrium was normal in size. The left ventricle was normal in both size and systolic function with an estimated left ventricular ejection fraction >60%.

**Figure 3:**
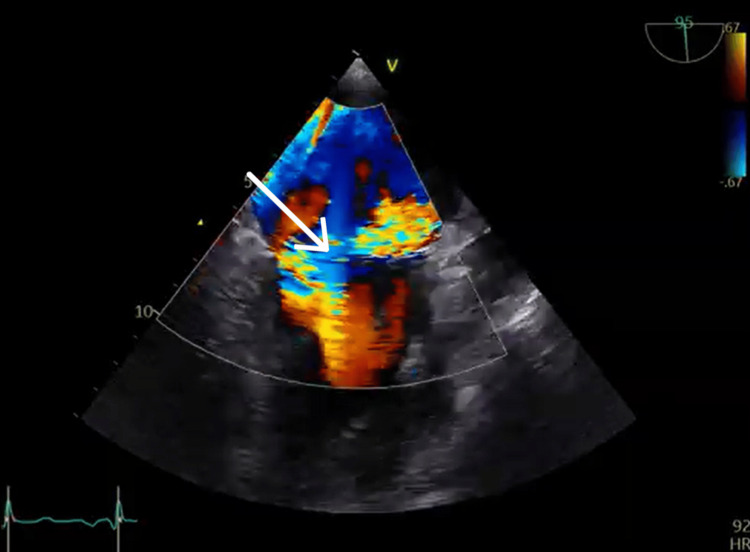
The transesophageal echocardiography revealed eccentric moderate-to-severe mitral regurgitation.

Flexible bronchoscope revealed positive hemorrhagic mucosa with initial BAL showing blood seven days after admission. In microbiology, the BAL culture was sterile.

As a result, a diagnosis of severe acute MR complicated by DAH was established. It was supported by the patient’s presentation of progressive dyspnea and pink-stained white sputum, alongside HRCT findings demonstrating diffuse, bilateral, asymmetric ground-glass alveolar opacities. Infectious and autoimmune etiologies were ruled out by sterile cultures and a negative autoimmune panel. A flexible bronchoscope further supported the diagnosis, revealing hemorrhagic mucosa and a bloody initial BAL. TEE confirmed the underlying mechanical etiology, demonstrating an acute rupture of the chordae tendineae. Therefore, the patient was scheduled for mitral valve replacement.

## Discussion

This case illustrates an unusual presentation of acute MR. A hypertensive and diabetic patient presented with DAH. Although previous cases have reported an association between DAH and chronic MR, this case describes DAH as the first presentation of acute MR, as the patient had no previous cardiac-related visits or admissions to the hospital. Moreover, a recent study that involved more than 715,000 diabetic patients found that type 2 diabetes has a positive association with aortic and mitral stenosis, but a significantly lower risk for MR [4]. Notably, our patient’s 12-year history of type 2 diabetes presents an epidemiological paradox; while diabetes is strongly linked to mitral stenosis, it is statistically associated with a lower risk of MR. This profile distinguishes our case from the existing literature [5-13].

An unexpected finding was the disproportionately low NT-proBNP level; while it is considered slightly elevated, it remained below the cut-off threshold for acute MR. Despite the limited availability of this false-negative phenomenon in the literature, it can be attributed to the inadequate stored peptides in the granules. The peptide is synthesized de novo in response to ventricular stress, leading to a temporal lag between symptom onset and the subsequent rise in laboratory results. Consequently, the level is spiking, but the initial blood sample might have been drawn too early to reflect the pathology. Compounding this, cases of acute MR may be associated with inadequate left ventricular wall stress, leading to a lack in the production of NT-proBNP [14]. Moreover, as the patient had hypertension for many years, the long-term regimen, specifically valsartan, may have aggravated this low value [15].

To understand the pathophysiology behind the occurrence of DAH, it is guided mainly by the effect of MR, including its risk factors and mechanical effects. Additionally, the initial presentation masked the actual diagnosis, thereby delaying the curative treatment.

Long-term hypertension plays a crucial role in increasing the risk of MR. It is associated with an increased risk of primary and secondary MR [16]. Although this may indeed increase the incidence of DAH secondary to acute MR, the major causes of acute MR are ruptured chordae tendineae (spontaneous or myxomatous degeneration), infective endocarditis, ischemic papillary muscle rupture, and trauma [17]. Within this differential diagnosis, infective endocarditis was effectively excluded based on negative blood cultures and TEE findings. The absence of trauma effectively excluded mechanical injury, while ischemic papillary muscle rupture was discounted based on a lack of prior myocardial infarction, an unremarkable electrocardiogram, stable troponin levels, and preserved left ventricular function. Consequently, spontaneous chordal rupture secondary to mitral valve prolapse emerged as the most probable etiology, a diagnosis strongly corroborated by TEE findings [18]. As a result, the development of acute MR leads to hemodynamic instability that causes a sudden increase in the left atrial volume, so it cannot adapt and comply with this marked increase, resulting in elevation of the left atrial pressure. After this hemodynamic deterioration, the pulmonary circulation leads to DAH and even pulmonary edema [13].

One of the diagnostic challenges was that pneumonia masked the diagnosis of DAH, so the patient was initially given regular pneumonia management. However, since there was suspicion of acute MR and no improvement despite receiving antibiotics, it required more diagnostic procedures, such as bronchoscopy and chest HRCT, which delayed the patient from undergoing the mitral valve replacement surgery, causing deterioration of his condition.

## Conclusions

This case highlights cardiac diseases as etiologies of DAH, demonstrating the importance of considering them as differential diagnoses of any unexplained DAH, especially in the presence of a pansystolic murmur, sterile cultures, and a negative autoimmune panel. Consequently, the early utilization of an echocardiogram is vital for identifying mechanical failures such as chordae tendineae rupture as the underlying cause, facilitating the rapid, life-saving intervention required to address acute hemodynamic compromise. In addition, this case serves as a critical warning against the isolated use of low NT-proBNP to exclude cardiac etiologies in emergency settings. Further large-scale observational studies are needed to explain this paradoxically low level despite the presence of acute mechanical valvular failure.
